# A Case of Aortopulmonary Window: Asymptomatic until the First Pregnancy

**DOI:** 10.1155/2015/935253

**Published:** 2015-09-17

**Authors:** Murat Kose, Serra Ucar, Samim Emet, Timur Selcuk Akpinar, Kıvanc Yalin

**Affiliations:** ^1^Department of Internal Medicine, Istanbul University Istanbul Medical Faculty, Turkey; ^2^Department of Nephrology, Istanbul University Istanbul Medical Faculty, Turkey; ^3^Department of Cardiology, Istanbul University Istanbul Medical Faculty, Turkey

## Abstract

The aortopulmonary window (APW) is an abnormal communication between the ascending aorta and the pulmonary trunk in the presence of two separate semilunar valves. It is a rare congenital malformation which represents 0.1% of all congenital cardiac diseases. Herein, we report a very rare case of 27-year-old patient with unrepaired APW causing Eisenmenger syndrome and pulmonary hypertension who was asymptomatic until her first pregnancy. The median survival of uncorrected APW is 33 years. Aortopulmonary window is a very rare congenital anomaly. To our knowledge, asymptomatic adult case has not been reported until now. APW should be considered in the differential diagnosis of the severe pulmonary hypertension also in adult patients.

## 1. Introduction

The aortopulmonary window (APW) is an abnormal communication between the ascending aorta and the pulmonary trunk in the presence of two separate semilunar valves. It is a rare congenital malformation which represents 0.1% of all congenital cardiac diseases [1]. Severe pulmonary hypertension, Eisenmenger syndrome, and congestive heart failure develop in the first months of life if the operation is delayed. The closure of the defect is contraindicated in patients with Eisenmenger syndrome [2]. Herein we report a very rare case of 27-year-old patient with unrepaired APW causing Eisenmenger syndrome and pulmonary hypertension who was asymptomatic until her first pregnancy.

## 2. Case Presentation

**Figure 1:**
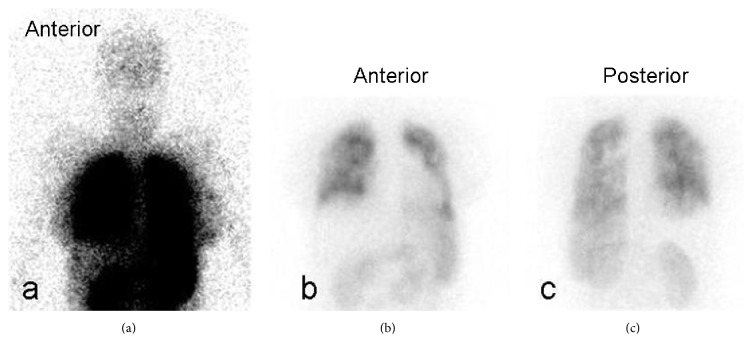
Images of pulmonary perfusion scintigraphy. Normal uptake in lungs was observed together with extrapulmonary uptake in cranium, spleen, and kidneys (a, b, and c) caused by systemic accumulation of Tc99m-MAA due to right to left shunt.

**Figure 2:**
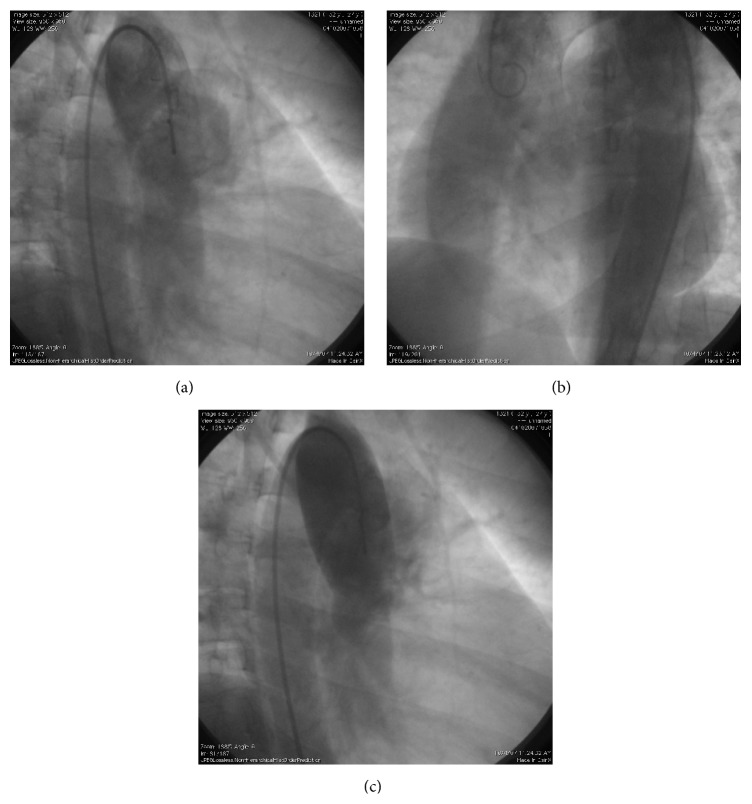
Aortic root injections, APW.

A 27-year-old female admitted to the emergency unit with severe dyspnea at the postoperative third day of the cesarean section. She expressed that she had no respiratory symptoms until the seventh month of her pregnancy; subsequently she developed exertional dyspnea. Because of intrauterine growth retardation, she gave birth in the 35th week of the pregnancy by Cesarean section. Physical examination on admission revealed tachypnea, tachycardia, and loud second heart sound. In room air, she had hypoxemia (PaO_2_: 51 mmHg) and hypocarbia (PaCO_2_: 28 mmHg) with oxygen saturation (SaO_2_) of 85%. D-dimer was 2281 *μ*g/L. Transthoracic echocardiography showed dilated right atrium and ventricle, tricuspid regurgitation, and normal left ventricular function with ejection fraction of 62%. Pulmonary artery pressure was 142 mmHg. Thorax computed tomography (CT) scan showed bilateral dilated pulmonary arteries and no sign of pulmonary thromboembolism. Oxygen and low molecular weight heparin were promptly started. On the third day of the treatment, SaO_2_ was 93% in room air. Hemoglobin was 16.2 g/dL, and hematocrit was 50.5%. Chest X-ray showed normal cardiothoracic index and bilateral dilated pulmonary arteries. ECG was in normal sinus rhythm with right axis deviation, incomplete right bundle branch block, and negative T waves between V1 and V4. Lower extremity Doppler ultrasonography (USG) was normal. No evidence of rheumatologic diseases could be found in her medical history. Rheumatologic markers were negative except speckled positive antinuclear antibody (ANA) with a titer of 1/160. Pulmonary perfusion scintigraphy was not diagnostic for pulmonary thromboembolism but showed systemic extrapulmonary accumulation of Tc99m-MAA in kidneys, spleen, and cranium which indicates right to left shunt (Figure 1). Transesophageal echocardiography was performed but did not reveal any additional findings to the transthoracic echocardiography. Right heart catheterization and aortic root injection showed a large APW and Eisenmenger syndrome with severe pulmonary hypertension with a pulmonary artery pressure of 131/32/97 mmHg and increased pulmonary vascular resistance of 9 Wood units/m^2^ (Figures 2(a), 2(b), and 2(c)). Vasoreactivity test with adenosine was negative. Because of the presence of Eisenmenger syndrome, closure of the APW was contraindicated. Endothelin receptor antagonist and warfarin were started. At follow-up, she was asymptomatic and SaO_2_ was 95% at rest; however she had dyspnea and became desaturated at exertion.

## 3. Discussion

APW is a very rare cardiac anomaly with right-left shunt which represents 0.1% of all congenital cardiac diseases [1]. Half of the patients with this disorder may also have other associated cardiac disorders such as atrial septal defect, patent ductus arteriosus, ventricular septal defect, coronary artery anomaly, and tetralogy of Fallot [1]. However our patient had an isolated APW. Patients with APW usually become symptomatic in the first month of life and the signs and symptoms show progression. Our patient had right to left shunting, severe pulmonary hypertension, and also Eisenmenger syndrome but no signs and symptoms of cardiac failure until 27 years old, that is, until the seventh month of her first pregnancy. On physical examination continuous cardiac murmur can be found approximately in half of the patients [3]. Our patient had no cardiac murmur but a loud second heart sound, especially at pulmonary area.

APW is associated with high mortality rates [1]. Pulmonary hypertension, Eisenmenger syndrome, and congestive heart failure develop rapidly. Therefore operation in early childhood is necessary [4]. APW also causes an increase in fetal and maternal mortality [5]. Our patient gave birth to a healthy baby with intrauterine growth retardation during the 35th week of pregnancy with no cardiac complications.

Echocardiography is an important technique for the diagnosis. In the literature, APW diagnosis is usually made by echocardiography and confirmed by catheterization [1, 6]. In our patient, both thoracic and transesophageal echocardiography could not detect APW. The diagnosis was established by right heart catheterization and aortic root injection.

Treatment consists of early correction of APW to avoid irreversible pulmonary hypertension. The surgical results are satisfactory when the APW presents as an isolated defect and when surgery is performed early [1]. However, surgery is contraindicated in patients with Eisenmenger syndrome [3]. Treatment of these patients includes anticoagulant drugs and phlebotomy sessions [7]. Warfarin and bosentan were prescribed to our patient because of symptomatic severe pulmonary hypertension. Phlebotomy was not needed to be performed because the hematocrit levels were less than 55% [8].

The median survival of uncorrected APW is 33 years [9]. An asymptomatic adult case has not been reported in the literature. There are two symptomatic patients, who had survival of more than 40 years. One of these patients was a female who lived until the age of 46 [10] and the other was a female who gave birth to three healthy children in her thirties and survived into her fifties with relatively preserved quality of life and died at the age of 60 [5]. Our patient had the first symptom when she was 27 years old. Until this age, she had a good quality of life without any respiratory and cardiac symptoms.

## 4. Conclusion

Aortopulmonary window is a very rare congenital anomaly. To our knowledge, asymptomatic adult case has not been reported until now. APW should be considered in the differential diagnosis of the severe pulmonary hypertension also in adult patients.
